# A guide to interpreting estimated median age of survival in cystic fibrosis patient registry reports

**DOI:** 10.1016/j.jcf.2017.11.014

**Published:** 2018-03

**Authors:** Ruth H. Keogh, Sanja Stanojevic

**Affiliations:** a Department of Medical Statistics, London School of Hygiene & Tropical Medicine, Keppel Street, London WC1E 7HT, United Kingdom; b Division of Respiratory Medicine, Department of Pediatrics, The Hospital for Sick Children, 555 University Avenue, Toronto, Ontario M5G 1X8, Canada; c Institute of Health Policy, Management and Evaluation, University of Toronto, 155 College Street, Toronto, Ontario M5T 3M6, Canada

**Keywords:** Annual report, Birth cohort, Conditional survival, Median survival, Patient registry, Period estimates, UK Cystic Fibrosis Registry

## Abstract

Survival statistics, estimated using data collected by national cystic fibrosis (CF) patient registries, are used to inform the CF community and monitor survival of CF populations. Annual registry reports typically give the median age of survival, though different registries use different estimation approaches and terminology, which has created confusion for the community. In this article we explain how median age of survival is estimated, what its interpretation is, and what assumptions and limitations are involved. Information on survival from birth is less useful for individuals who have already reached a certain age and we propose use of conditional survivor curves to address this. We provide recommendations for CF registries with the aim of facilitating clear and consistent reporting of survival statistics. Our recommendations are illustrated using data from the UK Cystic Fibrosis Registry.

## 1 Rationale

Survival statistics, estimated using data collected by national cystic fibrosis (CF) patient registries, are used inform the CF community and monitor survival of CF populations. Annual registry reports typically give the median age of survival, though different registries use different estimation approaches and terminology. Recently there have been efforts to standardize methodology and reporting [1], [2]. Nonetheless, there remain disparities in how survival is explained [3], [4], [5] (Supplementary Table 1), creating confusion for the community. Our aim is to provide clear descriptions of survival statistics and recommendations for reporting.

## 2 Median age of survival

Here we explain how median age of survival estimates are obtained, their interpretation, and the assumptions involved.

### 2.1 Survivor curves

A survivor curve shows the probability of living beyond a specific age for a range of ages. The median survival age is the age at which the survival probability is 0.5. Survivor curves are estimated from a mathematical formula that uses the proportion of individuals alive at each age who die before their next birthday (the ‘age-specific mortality rate’), or an extension of this which uses a finer time scale [6] (Supplementary materials S1–S3). In addition to using information on deaths, the calculations importantly also use the ages of people still alive.

**Fig. 1:**
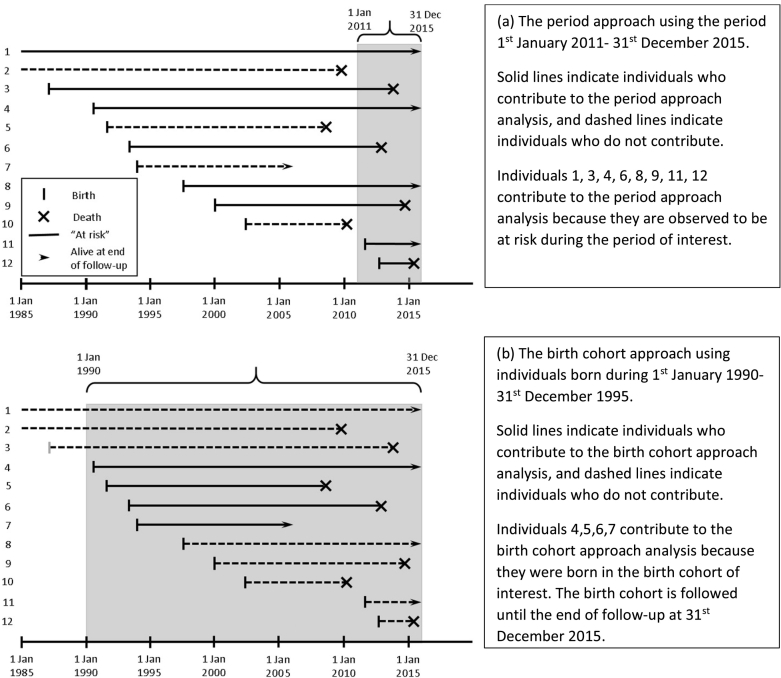
Illustrations contrasting the period approach and the birth cohort approach. Lines represent 12 individuals followed-up during 1st January 1985–31st December 2015. Individuals 1 and 2 were born prior to 1985 and individuals 1, 4, 8 and 11 were known to be alive at the end of follow-up. Individual 7 was lost-to-follow-up. Individuals 2 and 10 do not contribute to either analysis.

A survivor curve refers to the probabilities of living beyond specific ages from birth. To know the survivor curves for babies born today we would need to look into the future; instead, we must use information available now. There are two main ways of estimating curves: 1) the ‘period approach’ (a cross-sectional method) and 2) the ‘birth cohort approach’ (a longitudinal method). As illustrated in Fig. 1, these use different sets of individuals in the analysis, and are used to answer different questions. The period approach provides the most relevant information about predicted survival for babies being born with CF today, while the birth cohort approach is appropriate for comparing trends in survival in successive birth cohorts.

### 2.2 The period approach

This approach [7], [8], most often used by CF Registries, estimates a survivor curve using mortality rates at each age for people observed in the registry in a recent time period – usually the most recent 5-year period (e.g. 1st January 2011–31st December 2015). The mortality rate at each age is estimated by the number of people alive at that age during the 5-year period who died before their next birthday, divided by the total number of people alive at that age during the 5-year period. Most individuals advance through several ages during this period and hence contribute to the estimated mortality rate at several ages. Individuals do not contribute to estimated mortality rates for their ages before the 5-year period. The 5-year period is used to balance using data on a sufficient number of deaths for reliable estimates of age-specific mortality rates, and obtaining estimates that reflect current standards of care. The resulting estimated median survival age has the following interpretation:

*The estimated median survival age is the age beyond which we expect 50*% *of babies with CF born today to live*, *under the assumption that recent age*-*specific mortality rates will hold for the rest of their lives*.

Although period estimates of the survivor curve are obtained using people born at different times, we emphasize that the resulting survival statistics refer to a hypothetical cohort of babies born in the present day with CF and subject for the rest of their lives to current (2011–2015) age-specific mortality rates. The resulting estimates are based on an assumption that current mortality rates will apply in the future. This may be reasonable at younger ages, but less reasonable at older ages. For example, it assumes that the current mortality rate at age 30 will still apply 30 years from now, when babies born today would reach age 30. The estimates therefore do not account for potential impacts of future improvements in treatment.

### 2.3 The birth cohort approach

In this, survivor curves are estimated for a cohort of individuals born in a specified time period, e.g. 1st January 1990 to 31st December 1995. The birth cohort approach is useful for tracking the survival of successive cohorts of individuals with CF [9], [10]. However, estimates of survival from older cohorts are out-of-date for babies born with CF today, and recent birth cohorts cannot be used to estimate long term survival without extrapolation because median survival can only be estimated when 50% of the cohort have died [10].

## 3 Conditional survival

Information on survival from birth is less useful for individuals who have already reached a certain age. We therefore propose use of “conditional survivor curves” (Supplementary materials S4). The conditional survivor curve (Fig. 3) from age 30 shows the probability of survival beyond all ages greater than 30 for an individual aged 30. The probability of living beyond age 35 for an individual aged 30 is greater than the probability of living beyond age 35 from birth.

Under the period approach, the conditional median survival age has the following interpretation:

*The estimated conditional median survival age from age A is the age beyond which we expect 50*% *of individuals aged A today to live*, *under the assumption that recent age*-*specific mortality rates will hold for the rest of their lives*.

## 4 Other survival statistics

‘Life expectancy’ is the mean (or ‘average’) age to which individuals in a given population are expected to live from birth. However, survival ages tend to have a skewed distribution and life expectancy in CF can be strongly influenced by a few deaths at older ages. The mean survival age therefore tends to be higher than the median survival age. The median is generally agreed to provide a better summary statistic for survival ages. The median has the further advantage that it corresponds clearly to a position on the survivor curve and can be presented alongside other summary statistics based on the survivor curve, as illustrated in Fig. 1.

CF registry reports also often present the ‘median age of death’, which is obtained as the median of the ages at death of all individuals who died in a given year (e.g. 2015). The median age of death, as calculated in this way, does not consider the population still alive and does not provide a summary of survival. Unlike our recommended summary statistics of survival, this median age of death depends on the current age-distribution of the CF population. It is therefore not comparable across countries or over time, because of differences in age-distributions.

## 5 Recommendations and illustration

**Fig. 2:**
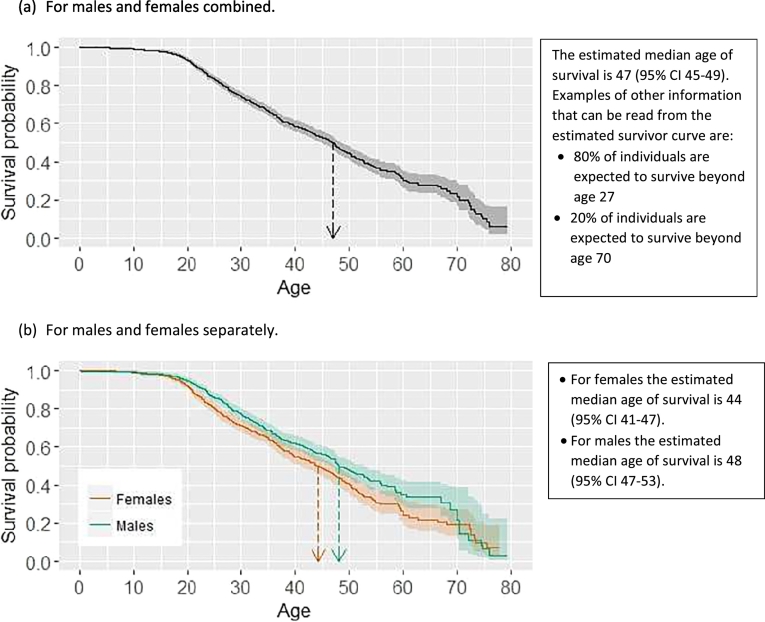
Estimated survivor curves using data from the UK Cystic Fibrosis Registry. The shaded area shows the 95% confidence intervals and the down-arrow indicates the estimated median survival age. Estimates are based on the period approach using data on 10,946 individuals and 654 deaths observed in the 5-year period 1st January 2011–31st December 2015.

**Fig. 3:**
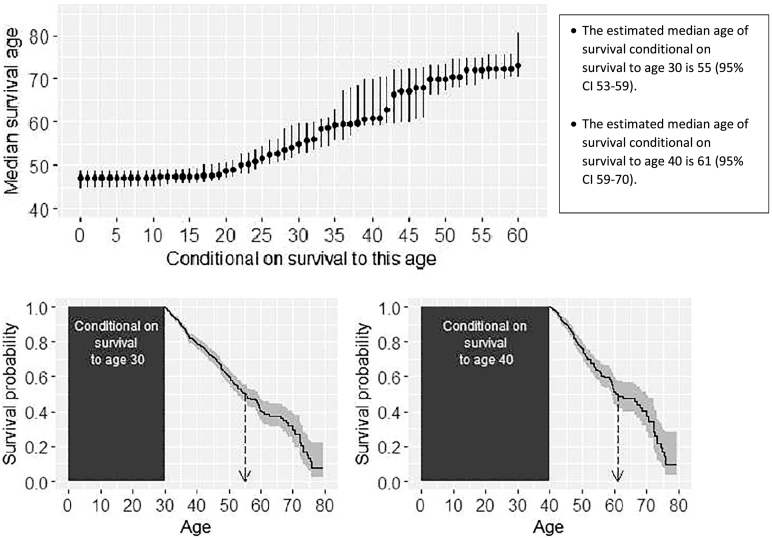
The upper plot shows estimates of median age of survival, conditional on survival to a given age. The dots show the estimated conditional median age of survival from each age and the bars show the 95% CIs. Estimates are based on the period approach using data on individuals observed in the 5-year period 1st January 2011–31st December 2015. The lower plots show the estimated conditional survival curves from ages 30 and 40 indicating the conditional median age of survival. Supplementary Fig. 1 shows estimates for males and females separately.

Table 1 and Fig. 2, Fig. 3 provide recommendations for reporting and presentation of survival statistics in annual registry reports, illustrated using data from the UK Cystic Fibrosis Registry. Supplementary Fig. 1 shows conditional estimates for males and females separately. All analyses are based on a period approach using the Kaplan-Meier method [6], and coincide with the analyses recommended by Sykes et al. [1]. For the period approach we used data on individuals observed during 1st January 2011–31st December 2015.

**Table 1:** Recommendations for presentation of survival statistics in national CF registry reports, illustrated using results from the UK Cystic Fibrosis Registry.

1. Present estimated survivor curves and the corresponding median survival age showing 95% CIs, overall (Fig. 2a) and for males and females separately (Fig. 2b), with the following text: *What is median age of survival*? *The estimated median age of survival is an estimate of the age beyond which we expect half of a population of individuals born today with CF in the UK to live*. *The interpretation of the median survival age of 47 is therefore that in a population of babies born today with CF half would be expected to die before the age of 47 and half would be expected to live beyond age 47*. *Limitations* *The estimation of the median age of survival assumes that age*-*specific mortality rates in the future will be similar to those today*. *Although it represents the best information available to us currently*, *it does not take into account recent and future improvements in care and treatment that will impact on survival*. *The median survival age refers to a population and not an individual*; *it does not take into account individual features, such as genotype, which impact on survival*. 2. Present estimates of median age of survival conditional on survival to a series of ages (Fig. 3), with the following text: *What is conditional median age of survival*? *The estimated median age of survival conditional on survival to age A is the age beyond which we expect 50*% *of individuals aged A in the present day to live*. *For example*, *the median age of survival conditional on survival to age 30 is 55*. *Therefore we expect 50*% *of 30 year olds with CF today to live beyond age 55*. *Limitations* *The limitations are similar to those for the overall median survival age*. *Conditional estimates do not take into account an individual*'*s current health status*, *which is highly informative about their survival prospects*.

We recommend that estimates are accompanied by confidence intervals [11], [12], which provide information about the degree of uncertainty in the estimates (Supplementary materials S5). Estimates from small registries may be subject to year-to-year fluctuations due to small numbers, and presentation of confidence intervals helps to avoid over-interpretation.

Ideally, individuals should be followed-up for death post-transplant in CF registries. Loss-to-follow-up due to transplant may result in biased survival estimates and reports should be clear on their handling of this [1], [2].

## 6 Conclusions

Survival statistics are valuable outcomes for registry reports. Standardized calculations and reporting aid interpretation and facilitate inter-country comparisons. We have focused on population-level survival. This does not incorporate individual features affecting survival, such as genotype [13], socioeconomic circumstances, or current health status, e.g. lung function. Further analyses can provide estimates of survival based on individual characteristics [14], [15], [16]. The methods we have described could in theory be applied in individual centres. However, the estimates would be expected to be subject to a large degree of uncertainty due to small numbers. Comparisons between centres should be made with caution, accounting for the features of the population served by the centre as well as this uncertainty. We have emphasized the limitation that survival estimates are based on an assumption that current mortality rates will hold in the future. There has been recent work to estimate trends in mortality rates in the US and UK and to make projections of future survivor curves based on different scenarios [13], [17]. National registries may consider providing such projections updated every 5 years or so. Further work is needed to study the potential impacts of targeted therapies on survival [18], [19].

## Appendix A Supplementary data

Supplementary Materials Image 1
