# Supplementary material for: A guide to interpreting estimated median age of survival in cystic fibrosis patient registry reports
Source: J Cyst Fibros. 2018 Mar;17(2):213–7. doi: 10.1016/j.jcf.2017.11.014 (PMC5885986; doi:10.1016/j.jcf.2017.11.014)
Supplement: Supplementary file 1 — Supplementary Materials [file mmc1.docx]

**Supplementary Materials**

**A guide to interpreting estimated median survival in cystic fibrosis patient registry reports**

**Ruth H. Keogh^a^, Sanja Stanojevic^b,c^**

^a^ Department of Medical Statistics, London School of Hygiene & Tropical Medicine, Keppel Street, London, WC1E 7HT, United Kingdom. [ruth.keogh@lshtm.ac.uk](mailto:ruth.keogh@lshtm.ac.uk).

^b^ Division of Respiratory Medicine, Department of Pediatrics, The Hospital for Sick Children, 555 University Avenue, Toronto, Ontario, Canada M5G 1X8.

^c^ Institute of Health Policy, Management and Evaluation, University of Toronto, 155 College Street, Toronto, Ontario, Canada M5T 3M6.

**S1. Definitions**

The survivor probability at age $t$, denoted $S(t)$, is the probability that an individual lives beyond age $t$. The survivor curve is a plot of the survivor probabilities across all the ages. At age 0 the survivor probability is equal to 1 (i.e. $S(0)$=1), because no individuals have yet died.

The age-specific hazard (or mortality) rate at age $t$, denoted $h(t)$, is the probability of death before age $t+1$ for an individual who has reached age$t$.

The median age of survival is the age $m$ at which $S(m)=0$.5

We focus on the period approach to estimating the survivor curve [4,5]. For illustration, we consider using data on individuals known to be alive at some time in a national CF registry between 1^st^ January 2011 and 31^st^ December 2015. This time period includes individuals who die during that period, individuals who are known to be alive at the end of the period, and individuals who become lost-to-follow-up during the period. Individuals who died before 1^st^ January 2011 are excluded, as are individuals who have been lost-to-follow-up prior to 1^st^ January 2011 and whose vital status is therefore uncertain. Individuals diagnosed after 31^st^ December 2015 are also excluded.

In the following descriptions, a ‘hat’ over a letter denotes an estimate, i.e. $\hat{h}(t)$ is an *estimate* of the age-specific hazard rate at age *t*, and $\hat{S}(t)$ is an *estimate* of the survivor probability at age *t*.

**S2. Life table estimates of the survivor curve**

The age-specific hazard rate at age $t$ can be estimated using

|  | $\hat{h}_{t}=\frac{d_{t}}{n_{t}}$ | (S1) |
| --- | --- | --- |

where $d_{t}$ is the number of individuals who die at age $t$ during 2011-2015 and $n_{t}$ is the number of individuals ‘at risk’ of dying at age $t$ during 2011-2015, which includes two groups of individuals:

1. individuals who have been observed to be at age $t$ during 2011-2015 but who did not die at that age.
2. individuals who died at age $t$ during 2011-2015.

In practice, some individuals may be lost-to follow-up at age $t$, meaning that they were last known to be alive some time before age $t$, but whose vital status at age $t$ has become uncertain. The definition of lost-to-follow-up may differ across registries depending on how their mortality data is obtained. For the UK Cystic Fibrosis Registry data, we recommend that individuals are first considered lost-to-follow-up two years after they were last observed in the Registry; the two years period is considered appropriate because further investigations showed that the majority of deaths are recorded in the Registry within two years of occurring. The use of a 2-year grace period also follows the recommendations of Sykes et al (2016). This is the approach taken for the example analyses presented in Figures 2 and 3 of the main text and Supplementary Figure 1. Different registries may decide to use a different length of time based on knowledge of their data collection procedures. We denote the number of individuals lost-to-follow-up at age$t$ by $l_{t}$. Accounting for potential loss-to-follow-up, the age-specific hazard rate at age $t$ can be estimated using

|  | $\hat{h}_{t}=\frac{d_{t}}{n_{t}-l_{t}/2}.$ | (S2) |
| --- | --- | --- |

The life-table estimate of the survivor probability at age t is

|  | $\hat{S}\left( t \right)=\prod_{k=1}^{t} \left( 1-\hat{h}_{k} \right)$ | (S3) |
| --- | --- | --- |

where $\hat{h}_{k} (k=1,\ldots,t)$ is as in equation (S2) and the product is over all ages up to and including age $t$.

**S3. Kaplan-Meier estimates of the survivor curve**

The Kaplan-Meier method [6] is similar to the life-table method but treats age continuously rather than in yearly (or more coarse) age groups. The Kaplan-Meier method therefore uses data at a finer level of detail than the life-table method, hence making the best use of the available individual-level data and providing more accurate estimated survival curves. We let $t_{1},t_{2},\ldots,t_{K}$ denote all of the exact ages at which an individual was observed to die during the period 1^st^ January 2011-31^st^ December 2015. An estimate of the hazard rate at exact time $t_{j}$ is:

|  | $\hat{h}_{t_{j}}=\frac{d_{t_{j}}}{n_{t_{j}}}$ | (S4) |
| --- | --- | --- |

where $d_{t_{j}}$ is the number of deaths at exact age $t_{j}$ (usually $d_{t_{j}}=1$) and $n_{t_{j}}$ is the number of individuals ‘at risk’ of death at exact age $t_{j}$, which comprises

1. individuals who are known to have been alive at exact age $t_{j}$ during 1^st^ January 2011-31^st^ December 2015. This excludes individuals who have been lost-to-follow-up prior to age $t_{j}$ and whose vital status is therefore uncertain at exact age $t_{j}$.
2. the individual(s) who died at exact age $t_{j}$.

The Kaplan-Meier estimate of the survivor function is

|  | $\hat{S}\left( t \right)=\prod_{t_{k}\leq t} \left( 1-\hat{h}_{t_{k}} \right)$ | (S5) |
| --- | --- | --- |

where the product is over all exact ages of death up to and including age $t$. Note that this is defined at any exact age $t$ (not just whole ages or observed ages).

**S4. Conditional survivor curves**

The survivor probability at time $t$ can be expressed mathematically as the probability $S\left( t \right)=Pr(T>t)$. A conditional survivor probability conditions on having already survived to a certain age. The probability of survival beyond age $t$ given survival to age $u (u<t)$ can be expressed as $S\left( t | T>u \right)=Pr\left( T>t | T>u \right)$. Using the laws of conditional probability, a conditional survivor probability is

|  | $S\left( t \vert T>u \right)=\frac{S\left( t \right)}{S\left( u \right)}$ | (S6) |
| --- | --- | --- |

The Kaplan-Meier estimate of the conditional survivor probability is

|  | $\hat{S}\left( t\vert T>u \right)=\frac{\prod_{t_{k}\leq t} \left( 1-\hat{h}_{t_{k}} \right)}{\prod_{t_{k}\leq u} \left( 1-\hat{h}_{t_{k}} \right)}=\prod_{{u<t}_{k}\leq t} \left( 1-\hat{h}_{t_{k}} \right)$ | (S7) |
| --- | --- | --- |

where the final product is over all observed death times between age $u$ and age $t$ (including $t$ but excluding $u$).

**S5. Confidence intervals**

Estimated survivor probabilities should be accompanied by confidence intervals (CI), which provide information about the degree of uncertainty in the estimate [7]. Typically 95% CIs are used. A 95% CI is an interval constructed in such a way that there is a 95% chance that this interval contains the true value of the parameter. A 95% CI also indicates the range of true values that the observed result is consistent with. Specifically, values outside the 95% CI would be contradicted (at the 5% significance level) by a hypothesis test whereas those inside the 95% CI would not be.

We give an interpretation using an example. The estimated survivor probability at age 30 is 0.74, with 95% CI (0.72,0.77). The interpretation of the estimate is that 74% of individuals are expected to live beyond age 30. The interpretation of the 95% CI is that the observed probability of 0.74 is consistent (considering hypothesis tests with p-value>=0.05) with true probabilities in the range 0.72 to 0.77 and inconsistent with values outside this range.

It is also possible to obtain 95% CI for the estimated median age of survival [8]. In the illustrative data from the UK, the estimated median age of survival is 47 with 95% CI (45, 49) (Figure 1(a)). The interpretation of the 95% CI is that the observed estimate of 47 is consistent (considering hypothesis tests with p-value>=0.05) with true values in the range 45 to 49 and inconsistent with values outside this range.

**Supplementary Table 1:** Extracts of descriptions of median survival taken from previous Cystic Fibrosis Registry reports from the UK (2015), US (2015) and Canada (2014). These extracts illustrate the differences in descriptions of median survival between the registries, and also show that previous descriptions included some incorrect statements.

| **UK Cystic Fibrosis Registry Report 2015 [1]**  Median predicted survival is a calculation based on people with CF recorded in the Registry as alive in the given year. A mathematical formula, which takes into account the age of those people in 2015, predicts how long we expect half of them to live. For 2015, this means that half of people registered as alive on the database are predicted to live to at least 45.1 years of age. Half of people alive today are currently predicted to die before they reach that age. |
| --- |
| **US Cystic Foundation Patient Registry Annual Data Report 2015 [2]**  Median Predicted Survival: All individuals currently in the Registry are included in this calculation. It reports the age at which 50 percent of the current population is expected to survive, given the current age distribution of the population and assuming that mortality rates do not change. |
| **The Canadian Cystic Fibrosis Registry 2014 Annual Report [3]**  Median age of survival is calculated based on cross-sectional data (i.e. data taken across different age groups) of the CF population and takes into consideration data from both individuals who have died AND those who are still alive. It is the estimated age beyond which 50 percent of the CF population would be expected to live, assuming the mortality rate in CF remained constant. This is NOT the age at which people with CF would be expected to die, (i.e. how long someone can expect to live, on average - see life expectancy above). Median age of survival is simply one way to evaluate survival in the CF population; however, there are other measures that provide us with additional information about how long people with CF are living (for example, median age at death and annual death rate).  When we say that the median age of survival in 2015 is 52.1 years, we are saying that if a child with CF is born in Canada in 2015, they have a 50% chance of living beyond 52.1 years of age based on current mortality rates. In other words, half of the CF population would be expected live to an age older than 52.1 years. Of course, mortality rates are not static and are constantly changing as new therapies and medicines for CF become available. Thus, this estimate is a reflection of the most accurate data that is available in 2015. |

**Supplementary Figure 1:** Estimates of median age of survival, conditional on survival to a given age for males and females separately. The dots show the estimated conditional median age of survival from each age and the bars show the 95% CIs. Estimates are based on the period approach using data on individuals observed in the 5-year period 1^st^ January 2011-31^st^ December 2015. The upper limits for confidence intervals were capped at age 85.

**
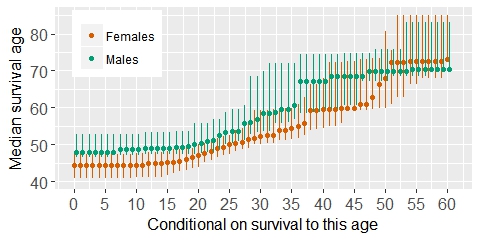
**

**References**

[1] Cystic Fibrosis Trust. Cystic Fibrosis Registry Report 2016. [https://www.cysticfibrosis.org.uk/the work-we-do/uk-cf-registry/reporting-and-resources](https://www.cysticfibrosis.org.uk/the%20work-we-do/uk-cf-registry/reporting-and-resources).

[2] Cystic Fibrosis Foundation. Cystic Fibrosis Foundation Patient Registry Annual Data Report 2015. <https://www.cff.org/Our-Research/CF-Patient-Registry/2015-Patient-Registry-Annual-Data-Report.pdf>.

[3] Cystic Fibrosis Canada. The Canadian Cystic Fibrosis Registry 2015 Annual Report. <http://www.cysticfibrosis.ca/news/publications>.

[4] Brenner H, Gefeller O. Deriving more up-to-date estimates of long-term patient survival. *J Clin Epidemiol* 1997; 50: 211-216.

[5] Smith LK, Lambert PC, Botha JL, Jones DR. Providing more up-to-date estimates of patient survival: A comparison of standard survival analysis with period analysis using life-table methods and proportional hazards models. *J Clin Epidemiol* 2004; 57: 14–20.

[6] Kaplan EL, Meier P. Nonparametric estimation from incomplete observations. *J Am Stat Assoc* 1958; 53: 457-481.

[7] Kalbfleisch JD, Prentice RL. The statistical analysis of failure time data, 2^nd^ edition. John Wiley & Sons, New York. 2002.

[8] Brookmeyer R, Crowley J. A Confidence Interval for the Median Survival Time. *Biometrics* 1982; 38: 29-41.
